# Guild structure, diversity and succession of dung beetles associated with Indian elephant dung in South Western Ghats forests

**DOI:** 10.1673/2006_06_17.1

**Published:** 2006-09-05

**Authors:** Thomas K. Sabu, K. V. Vinod, P. J. Vineesh

**Affiliations:** P.G. and Research Department of Zoology, St. Joseph’s College, Devagiri, Calicut, Kerala, India 673008

**Keywords:** Catharsius sagax, Copris davisoni, Copris repertus, Copris signatus, Drepanocerus setosus, Gymnopleurus melanarius, Heliocopris dominus, Liatongus indicus, Oniticellus cinctus, Onitis siva, Onitis subopacus, Onthophagus bronzeus, Onthophagus cervus, Onthophagus ensifer, Onthophagus falsus, Onthophagus furcillifer, Onthophagus madoqua, Onthophagus rectecornutus, Sisyphus longipes, Sisyphus neglectus, species richness, succession

## Abstract

The diversity, guild structure and succession of dung beetles associated with Indian elephant dung is described in a deciduous forest site in Western Ghats, a hot spot of diversity in India. Dung beetles were collected using baited pitfall traps and from exposed dung pats in the forest at intervals of 1, 3, 5, 7, 15 and 21 days. Twenty-one dung beetle species belonging to the 3 major functional guilds were recorded. Abundance of dwellers was high compared to rollers deviating from earlier reports on the high abundance of rollers in the afrotropical regions. Dweller Drepanocerus setosus and tunneler Onthophagus bronzeus were the most abundant species. Dung pats aged 3–5 days attracted the highest abundance of dung beetles. Bray Curtis similarity index indicated low community similarity between different stages of succession. Species richness and abundance of tunnelers increased with dung age and decreasing moisture up to a threshold level, followed by a decrease. Rollers and dwellers did not show any significant relationship with dung moisture content. Further research is needed to estimate the dung beetle community associated with the dung pats of other mega herbivores as well as of elephant dung in other forests of the Western Ghats.

## Introduction

**Figure 1:**
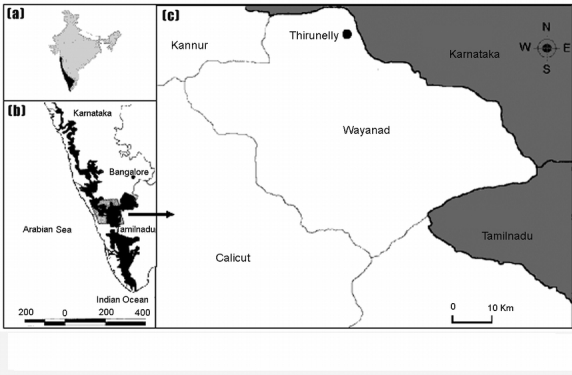
(a) Map of India showing the location of the Western Ghats. (b) The Western Ghats. (c) The study site in the Wayanad region of the Western Ghats.

The Western Ghats, fringing the Arabian Sea coastline of the Indian peninsula (Fig. 1), contains one of India's last remaining areas of tropical rainforest (WWF 2001) and is a listed hot spot of biodiversity (WCMC 1992;Myers 1988). Elephant (Elephas maximus Linnaeus), gaur (Bos gaurus H. Smith), sambar deer (Cervus unicolor Kerr) and spotted deer (Axis axis (Erxleben)) are the major mammalian herbivores in the moist deciduous and evergreen forests of the Western Ghats (WWF 2001). Two types of dung beetle communities are expected to be found on the floor of these forests: those associated with the larger dung pats of elephant and gaur, and those associated with the smaller droppings of deer and boar. Dung beetles participate in the recycling of matter, nutrients and energy, and contribute to the natural process of regeneration of the ecosystem. Many species occur in large populations, and dung beetles occupy diverse ecological niches due to their dung foraging tactics and life history traits. Since dung beetles depend generally on dung produced mainly by mammals, such resource can be extremely patchy in space and time. Hence, resource partitioning and competition are prime features of dung beetle species assemblages (Hanski 1991a).

Though many entomological expeditions have visited the region and a systematic list of the dung beetles is available (Balthasar 1963 a, b; Arrow 1931; [Bibr i1536-2442-6-17-1-Biswas1][Bibr i1536-2442-6-17-1-Biswas2]; Chatterjee and Biswas 1995, 2000;Biswas and Mulay 2001) no studies have addressed the ecology, community structure and succession pattern of the dung beetles associated with the herbivorous mammals of the region. By contrast, the community structure, diversity and abundance, successional trends, diurnal activities and resource partitioning of dung beetle communities associated with the dung pats of large herbivores in afrotropical regions are relatively well documented (Cambefort 1991; Cambefort and Walter 1991; Cambefort and Hanski 1991; Doube 1991).

Dung beetles exploit patchy and ephemeral dung pats as resources and hence strong competition between co-occurring species plays a major role in structuring communities (Feer and Pincebourde 2005). Dung beetles are divided broadly into 3 functional groups based on their nesting strategies viz., rollers (telecoprid nesters), tunnelers (paracoprid nesters) and dwellers (endocoprid nesters) (Cambefort and Hanski 1991). Rollers form balls from a dung pat, which are rolled away, buried and used for feeding and breeding. Tunnelers create underground chambers beneath the dung pat and construct their nests using the dung from the pat whereas the dwellers breed in the dung pat itself (Cambefort and Hanski 1991; Holter *et al.* 2002). This functional stratification allows dung beetles to minimize the intense competition for limited food and space and also to protect the food from adverse environmental conditions. Numerous reports are available on resource competition between dwellers, tunnelers and rollers as entire droppings can be removed or shredded by beetles within a few minutes (Hanski andCambefort 1991). Based on the order of arrival and rate of resource utilization of dung pats, dung beetles are grouped into type I and type II categories (Hanski 1991b). Type 1 beetles are superior competitors, adapted to use fresh dung as fast as possible while Type II species are adapted to use older dung.

Age, size, water content and texture of the dung matter determine the preference of dung beetles towards a specific dung type (Anderson and Coe 1974; Sowig and Wassmer 1994). In ruminant dung, the surface crust prevents the loss of moisture from the bottom layers and provides the needed moisture for a prolonged stay of some beetle groups (Hanski 1980), however, in elephant dung no such crust formation takes place (Anderson and Coe 1974). Some dung beetles prefer the coarse-textured dung of elephants while others prefer the more fluid and fine-textured dung of ruminants (Holter *et al.* 2002).

The aim of this work is a preliminary analysis of the diversity, guild structure and successional trends of dung beetles in relation to dung exposure time and moisture in the dung of Indian elephants in the deciduous forests of Wayanad region of South Western Ghats.

## Materials and Methods

### Study area

The work was carried out in the Thirunelly forests (20.55 km^2^) located in the northern boundary of South Western Ghats forests in Wayanad in the Nilgiri Biosphere region [5520.4 km^2^], 100 km North of Calicut, Kerala state (Fig. 1). The Nilgiri Biosphere region harbors a population of 2990 elephants (Sukumar 1989). Vegetation of this region consists of patches of deciduous and evergreen forests. A 2.5 km^2^ area was selected as the study site. Biogeographically, the Wayanad region of the Western Ghats is a transitional zone between the moist forests of the South Western Ghats and dry forests of the northern region. This habitat harbors restricted, endemic species as well as disjunct populations of species that are found in both regions (Pascal 1988; WWF 2001). The northeast monsoon from October to November supplements the June to September southwest monsoon rainfall. Because of the deeply dissected topography, this area receives 3000–3500 mm of rainfall throughout the year. This is in contrast to nearby areas, which receive 5000–6000 mm of rainfall from the moisture-laden southwest monsoon winds from the Malabar Coast (Mishra and Johnsingh 1998; Forests and Wildlife Department Working Plan 2001). This variation in rainfall and topography has led to local variations in habitat types and the presence of localized centers of endemism (Rodgers and Panwar 1988; Kenderick 1989). Deciduous forests in the region are the preferred foraging area for herds of elephants and gaurs arriving from the comparatively drier Deccan region during the post- rainy seasons as the adjoining open grasslands in the upper ranges together with the abundance of bamboo culms (Bambusa sp.) provide a wide choice of resource materials for grazing and browsing (Joy 1991, Nair 1991).

### Methodology

Two approaches were used for sampling. One estimated the dung beetle guild structure, diversity and abundance, and the other estimated the successional trends of dung beetles in relation to dung age and moisture. To analyze the diversity and guild structure of beetles attracted to elephant dung bait-surface-grid pitfall traps were used (Lobo *et al.* 1988;Veiga *et al.* 1989).

Field studies were conducted during the post-rainy period of November – December, 2003. 10 bait-surface-grid pitfall traps made from plastic basins (21cm in diameter, 15 cm deep) were set into the ground at randomly selected spots separated by about 500 m which were close to elephant tracks. The top of the basins was even with the level of the surrounding substrate and a water-formalin-liquid soap mixture was added as preservative. A 25 x 25 x 2 cm plastic board supported by 15 cm iron poles was set over each trap to protect it against debris and rain. One kg of bait was used in each trap as larger insects have a preference towards large droppings and larger baits (Peck and Howden 1984). The trap contents were collected three times at weekly intervals, taken to the lab in polythene bags, the beetles were separated, preserved in 70% ethanol and were sorted into species from each sample. Examples of species from each sample were mounted and labeled. Species identification was done by the authors and with the assistance of specialists (see Acknowledgements). Specimens are temporarily curated in the insect collections of St. Joseph’s College, Devagiri, Calicut, and will be transferred to the Zoological Survey of India, Western Ghats regional station, Calicut.

A combination of dung pat and pitfall traps was used to analyze the succession of dung beetles in the elephant dung pats in the forest site. Pitfall traps were used in order to trap rollers, which would have otherwise moved away from the dung (unlike dwellers and tunnelers). Thirty-six fresh dung pats were collected in polythene bags from the study area between 6 am to 10 am during the study period on a single day. Pats are well suited for studies of patterns of resource partitioning because all beetles can be extracted completely from a chosen feces pat (Sowig and Wassmer 1994). To limit the bias arising from the influence of patch size on the dung beetle communities, pats of equal weight were used (Holter 1982; Sowig and Wassmer 1994). Fresh dung was differentiated by the wetness, heat and the pungent smell during non-rainy periods. Forest trackers of resident tribal communities helped to spot fresh dung pats. There were no other effective methods to collect fresh dung from these forests that are locally well known for frequent man-elephant confrontations. Spotting elephants from a safe distance is practically impossible due to the uneven terrain and the presence of thick under storey of bamboo culms (Bambusa arundinaceae) and shrubs (Abutilon persicum, Helecteris isora, Lantana camera etc.).

Six sets, each consisting of five dung balls positioned 50 cm from each other, were placed in randomly selected spots in the forest floor 100 m apart. Dung balls were fenced with bamboo twigs to prevent disturbance by birds. Six bait-surface-grid traps baited with fresh dung were placed 50 cm apart around each set of dung balls to trap the rollers. Thus a total of 30 dung balls surrounded by 30 traps were set in 2.5 km^2^ of area. To avoid the influence of bait size on rollers (Sowig and Wassmer 1994), each bait was prepared by partitioning a dung ball into 6 portions. Dung balls and the associated pitfall traps of each set were collected at intervals of 1, 3, 5, 7, 14 and 21 days. Each dung ball used in the successional study was separated into an upper layer of dung matter and a bottom layer of dung mixed with soil. Beetles from both layers were sorted separately by floatation methods (Moore 1954). This enabled the separation of dwellers in the pats and the tunnelers below the pats. Further, tunnels deep beneath the pats were followed with a spade to collect the tunnelers. Collected beetles were transferred to 70% alcohol, identified and categorized into different nesting guilds based on field observations and the literature (Holter *et al.* 2002;Cambefort and Hanski 1991). Twenty grams of dung from each dung ball group was collected, sealed in glass tubes, and oven dried at 105° C until reaching a constant weight. Moisture content was expressed as percentage wet weight (Anderson and Coe 1974).

### Data analysis

All diversity analyses were based on pit fall trap collection. Species diversity was calculated using Fisher’s alpha diversity index (α) (Fisher *et al.* 1943), Simpson dominance index (D) (Simpson 1949) and E_var_ evenness index (Smith and Wilson 1996). Bray Curtis similarity index (Bray and Curtis 1957) after 4^th^ root transformation was used to compare the similarity between the assemblages arriving at different times on the dung pats followed by cluster analysis. The 4^th^ root transformation is ideal for samples with abundant and rare species as this leads to the down weighting of abundant species and allows the midrange and rarer species to exert influence on the calculations of similarity (Clarke and Warwick 2001). Species richness was predicted using non-parametric (Chao1, Chao2, ACE, ICE, Jack1, Jack2 and Bootstrap) and parametric (Michaelis-Menten means) species richness estimators (Colwell 2000, Colwell and Coddington 1994). For every estimator, bias was calculated as the mean proportional deviation of the estimate S_est_ from S_true_ (Brose and Martinez 2004). E_var_ was computed with the program Gleason’s software (version 2004). Species richness estimators, Fisher’s alpha diversity index and Simpson dominance index were calculated with EstimateS software (Colwell 2000). Bray Curtis similarity index was analyzed with Primer 5.

Scatter plots of species richness, abundance of the community and the three nesting guilds against moisture content and dung exposure time were plotted using data from succession studies. Richness and abundance of the community and tunnelers indicated a change in the slope of the function on the third day of dung exposure at a critical moisture level, which was taken as the threshold dung age and moisture level. Abundance of dwellers displayed a linear relationship while species richness of dwellers, and abundance and richness of rollers showed no relationship with dung exposure time and moisture content. Hence, a piecewise linear regression model was employed to analyze the relationship between richness and abundance of the community and tunnelers with dung exposure time and moisture; and simple linear regression analysis was used to analyze the relationship between abundance of dwellers with dung exposure time and moisture content (Zar 2003; Gujarati 2003). All statistical analyses were performed using Gretl software (version 1.3.3).

## Results

### The dung beetle community guild structure, diversity and abundance

A total of 309 individuals representing 21 species, 10 genera, six tribes and three nesting guilds were collected (Table 1). The tunneler guild (15 species) was the most species rich (71.4%) and abundant (57.6 %) of the dung beetle guilds, and rollers were the least abundant guild (9.7%). D. setosus (23.3%), followed by O. bronzeus (18.8%), Onthophagus cervus (18.1%) and Liatongus indicus (9.61%) were the most abundant species in both pitfall traps and the successional set ups.

**Table 1:**
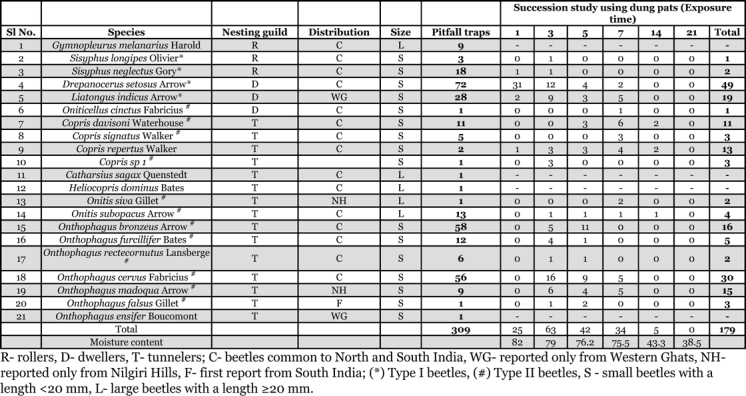
Diversity, abundance, guild structure and succession of dung beetles associated with Indian elephant dung.

The assemblage is moderately diverse (α= 5.09) and with low evenness (E_var =_ 0.27) (Table 2). Mean diversity by Fisher’s alpha diversity index was highest for tunnelers (α= 3.9), mean evenness by E_var_ was high for rollers (E_var_ = 0.68), and mean dominance by Simpson dominance index was highest for dwellers (D = 0.58).

**Table 2:**
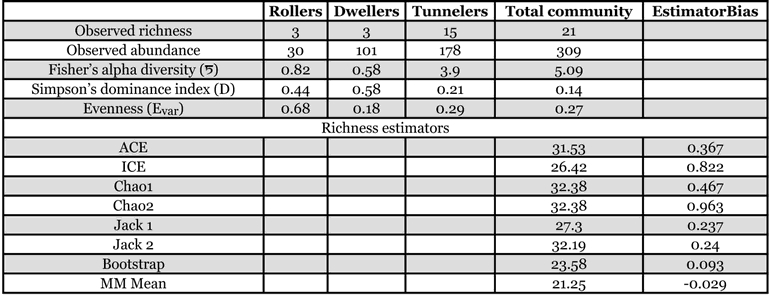
Richness estimators, diversity and dominance indices of the dung beetle species associated with elephant dung.

**Figure 2:**
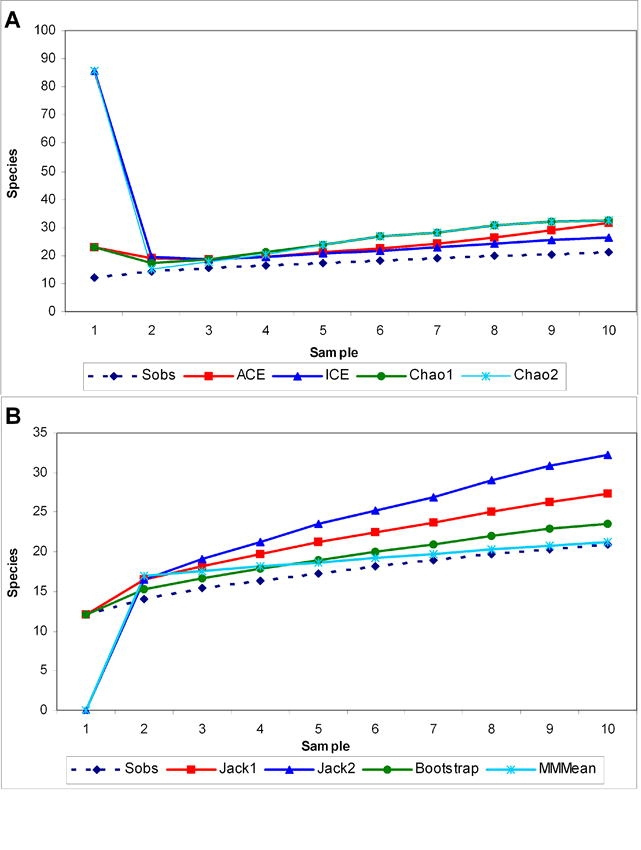
A, B. Randomized species accumulation curves of dung beetles attracted to baited pitfall traps from South Western Ghats deciduous forest at Thirunelly.

Except for Bootstrap and MM means all other species richness estimators overestimated the species richness and indicated the occurrence of an additional 5–11 species (Table 2). All estimators recorded bias in the range of 0.09–0.96. Estimator bias was highest for Chao 2 and ICE; and lowest for MM mean and Bootstrap. None of the estimators reached an asymptote with observed species richness (Fig. 2 A, B).

### Successional trends

179 dung beetles representing 17 species, seven genera and five tribes were present during the succession study (Table 1). Heliocopris dominus, Gymnopleurus melanarius, Catharsius sagax and Onthophagus ensifer,which were present in the pitfall traps were not reported from the collections for succession studies.

**Figure 3:**
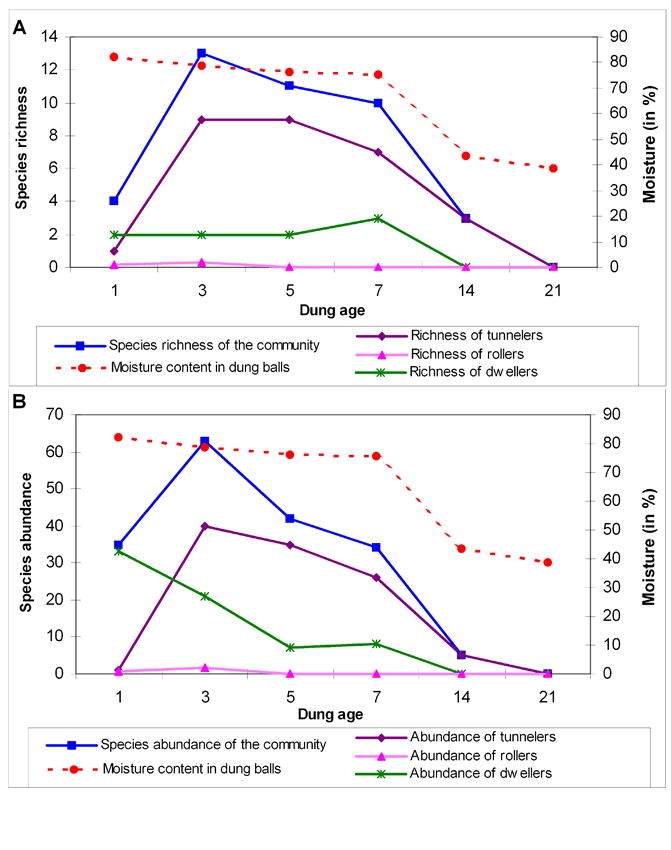
Relationship between species richness (A) and abundance (B) of dung beetles and moisture plotted against dung age.

Dung moisture content decreased with progressing dung exposure time in two phases (Fig. 3 A, B); a slow decline during 1–7 days (6.7%) and steep decline from 7–14 days (32.2%). During the 1^st^ phase, richness and abundance of the beetle assemblage and the tunneler nesting guild increased up to the 3^rd^ day followed by a gradual fall, which accelerated during the second phase (Fig. 3 A, B). Dweller abundance declined in relation to moisture and dung exposure time but richness did not show any relationship with either parameter.

Richness of the dung beetle assemblage significantly increased up to a threshold moisture level of 78.8 % (β_1_ = –2.339, p < 0.01) on the third day (β_1_ = 4.309; p < 0.01). Subsequently, richness declined significantly in relation to moisture content and dung exposure time (β_1_+ β_2_= 0.278, β_1_+ β_2_= −0.746, p < 0.01). Abundance did not show any significant relationship with moisture content (first phase β_1_ = −4.169, second phase β_1_+ β_2_ = 1.247, p = ns) and dung exposure time (first phase β_1_ = 8.561, second phase β_1_+ β_2_ = −3.332, p = ns). Analysis of the relationship between species richness and abundance of nesting guilds with moisture content and dung exposure time showed three contrasting patterns. Species richness and abundance of tunnelers were significantly related to moisture content and dung exposure time: both increased with dung exposure time (β_1_= 4.161, p < 0.01; β_1_= 18.261, p < 0.05 for richness and abundance respectively) and moisture levels (β_1_= −2.287, p < 0.05; β_1_= −10.167, p < 0.01 for richness and abundance respectively) up to a threshold level and subsequently decreased (β_1_+ β_2 =_ −0.532, p < 0.01; β_1_+ β_2_ = −2.332_,_ p < 0.05 for richness and abundance respectively with exposure time, β_1_+ β_2_ = 0.194, p < 0.05; β_1_+ β_2_ = 0.879_,_ p < 0.01 for richness and abundance respectively with moisture content). As per linear regression analysis, abundance of dwellers declined steadily in relation to moisture content (β= 0.512, p = ns) and dung exposure time (β= –1.390, p = ns) without any significant relationship. Since rollers were present only in fresh dung and absent after the initial days of succession, no specific relationship could be determined for this group.

Based on the order of arrival, all beetles except Copris repertus were grouped into type I and type II categories (Table 1). Type 1 consisted of all rollers and 2 dwellers and the type II consisted of all tunnelers and one dweller. Copris repertus could not be placed under neither categories as it was present in both old and fresh dung pats. Based on the dominance of nesting guilds, the succession of beetles in dung pats were categorized into 3 phases. Dwellers dominated the initial phase (1–3 days), tunnelers consisting of predominantly of Onthophagus species dominated the second phase (3–5 days) and tunnelers belonging to Copris species dominated the final phase (7–14 days) of succession. Presence of both dwellers and tunnelers lead to the high abundance on the 3^rd^ day of succession. Two species were exceptions with C. repertus being present throughout the 3 phases of succession and Onitis subopacus during the last two phases.

**Figure 4:**
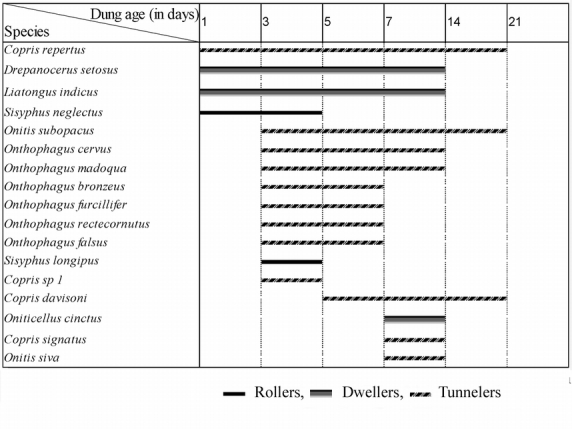
Succession pattern of dung beetles associated with elephant dung in the South Western Ghats forest at Thirunelly.

Time until first arrival and the duration of stay varied across species and the three nesting guilds (Fig. 4). Rollers with two species were the least speciose and least abundant among the three guilds and were present for a short period of only 1–3 days. The presence of dwellers was limited to the period of high moisture content in the dung (82–75%) (1–7 days). Their abundance and richness increased in relation to dung age and moisture till 7^th^ day after which they were not recorded. Drepanocerus setosus and L. indicus arrived on first day and Oniticellus cinctus on the 7^th^ day of succession. Tunnelers were present in the pats from 1^st^ day to 14^th^ day. Their abundance and richness increased up to 3^rd^ day followed by a gradual decline in relation to dung age and moisture loss till 7^th^ day, followed by a steep decline. Members of Onthophagus and Copris dominated the tunneler guild. Onthophagus species were present from days 3–7 at a time of high dung moisture (79–75%). Copris species displayed no specific pattern either in the arrival pattern or duration of stay.

**Figure 5:**
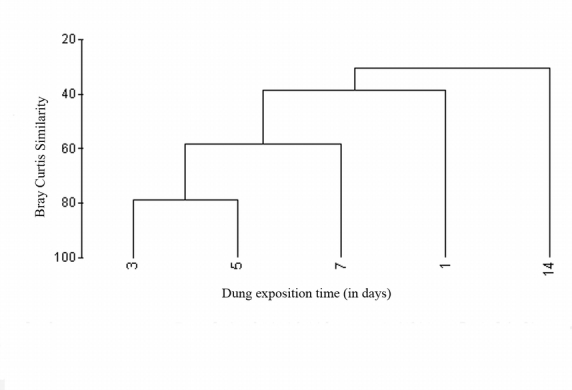
Dendogram based on hierarchical agglomerative clustering (group-average linking) showing the similarities between the dung beetle assemblages present during different stages of succession.

A distinct pattern of dung resource partitioning within the tunneler guild was present. Onthophagus species dominated the fresh dung of 3–5 days of dung exposure; Copris signatus, Copris davisoni and Onitis siva dominated the old dung from the 7^th^ –14^th^ day, and a third group of C. repertus and O. subopacus was present in both fresh and old dung without any dung age preferences during 3–14 days. The beetle assemblages present during 3–7 days of succession was the most similar, and assemblages present during the final and initial days of succession were least similar (Fig. 5).

## Discussion

Analysis of the community structure of dung beetle assemblages associated with elephant dung pats in the study region showed high species richness and abundance of tunnelers and low richness and abundance of rollers and dwellers. These findings are in agreement with the earlier reports (Hanski and Krikken 1991) on the guild composition in larger herbivore dung from South East Asia region and about the rarity of rollers from Bengal in India (Oppenheimer 1977). The abundance of small rollers (Sisyphus) and the low presence of large rollers (Gymnopleurus) in pitfall traps and succession studies indicated the dominance of smaller rollers in elephant dung of the region, contradictory to previously published general trend of the association of large rollers with bigger dung pats (Hanski and Krikken 1991). Similarly, the high abundance of the dweller, D. setosus, in baited pitfall traps and succession studies is differing from the reported low presence of dwellers in the dung beetle assemblages in the tropical region (Hanski and Krikken 1991). Our studies with baited pitfall traps and floatation methods to analyze the dung beetle diversity of the Wayanad forests (as part of a broader project on forest floor insect diversity) showed very low abundance of G. melanarius and high abundance of D. setosus in the region. The low evenness of the assemblage is linked to the dominance of three species, namely D. setosus, O. bronzeus and Onthophagus cervus.

Indirect criteria for a good species richness estimator are that the estimator curve should reach an asymptote with fewer samples than are required for the observed species accumulation curve, and there should be a reasonable visual extrapolation of the asymptote of the observed species accumulation curve with the estimator curves (Toti *et al*. 2000). The estimator should also be sensitive to the number of rare species and to the patchiness of the samples (Chazdon *et al.*. 1998; Magurran 2003). Our analysis did not meet either of these criteria, which indicates the inadequacy of our sampling effort. The two exceptions to this are derived from the MM mean (which produces stable estimates with a small number of samples) and the sample size-sensitive Bootstrap estimator (Magurran 2003). The other six estimators predicted the presence of an additional 5 –11 species in the elephant dung.

Abundance of dwellers and a lower abundance of rollers were noticed in the early stages of succession. The lower abundance of rollers in fresh dung is related to the dominance of dwellers in the dung balls as the dweller activity makes it difficult for rollers to form balls (Heinrich and Bartholomew 1979). But whether it is applicable to the situation in the Thirunelly forests demands further analysis. Abundance of D. setosus in fresh dung pats indicate *s* their ability to locate fresh pats before other guilds and their preference towards fresh dung. We attribute the sudden decline of D. setosus starting on the 3^rd^ day of succession to the inference from the late arriving tunnelers. Though D. setosus are known for their prolonged stay in larger pats for longer periods (Cambefort 1991) their sudden decline from 3^rd^ day of succession, we attribute to the inference from the late arriving tunnelers. Similarly their presence up to the seventh day of succession prompts us to consider that it is competitive exclusion (Sowig and Wassmer 1994) of the early arriving dominant dwellers by the late arriving tunnelers rather than dung age and moisture which is leading to their decline after the third day. Sequential distribution and resource partitioning by the three dweller species along the fresh dung niche axis was distinct; D. setosus dominated the fresh dung, L.indicus dominated on the 7^th^ day and Oniticellus cinctus arrived on the 7^th^ day. Presence of all dwellers during the high moisture regime (1–7 days) indicates their preference towards moist dung.

Tunnelers avoided the temporal overlap of species and competition between the members of the guild by arriving in the pat at different times. Based on their order of arrival in relation to dung age and duration of stay in the pats, tunnelers were categorized into three groups. The first group consisted of predominantly Onthophagus species and was present on the 3^rd^ and 5^th^ days; their massive appearance lead to the steep rise in the dung beetle population on 3rd day and the competitive exclusion of the earlier settlers. Increased tunneling activity and quick removal of dung materials during the brief stay of tunnelers makes the dung porous and the resulting loss of moisture leads to a steady decline in the abundance and richness of beetles (Anderson and Coe 1974). Hence, the decline in the dung moisture loss and the steep fall in beetle abundance in dung pats since the 7^th^ day of succession was related to the massive arrival of Onthophagus and Copris species in the dung pats. The second group consisting of O. siva, C. signatus, and Oniticellus cinctus were the last arrivals on dung pats on 7^th^ day. Onitis are tunnelers which prefer old dung (Hanski 1980) and we included C. signatus in this category. The third group of C. repertus and O. subopacus was present both in old and fresh dung. Presence of the larger 2^nd^ and 3^rd^ groups in the less crowded old dung pats indicated that both these large tunneler beetle species have the capacity to utilize undigestible larger particles in elephant dung. This contrasts with the smaller tunnelers and dwellers which prefer smaller and more nutritious particles, including dead epithelial cells of elephant gut, in dung pats (Holter *et al.* 2002). This statement is speculative and further analysis is required on the feeding preference of large tunnelers towards dung of different ages and the impact of the exclusion of Onthophagus on dung pats.

The order of arrival and distribution of the two most speciose genera, Copris and Onthophagus, along a niche axis in the pats showed two contrasting patterns. Copris species arrived at different stages of dung succession and all Onthophagus species arrived on the 3^rd^ day of succession. It appeared that one set was following Gause’s principle (Gause 1934) of the non-occurrence of closely related species in a habitat and the other the opposite trend.

Coexistence of closely related Onthophagus species for a shorter period in the pats is attributed to the preference of Onthophagus towards a high moisture regime in dung pats (Sowig and Wassmer 1994), which leads to their coexistence together for a shorter duration in the ephemeral microhabitats. It is another instance of species with ecologically similar demands coexisting in unstable patchy environments (Shorrock 1990; Sowig and Wassmer 1994). Richness and abundance of tunnelers are strongly correlated with dung age and moisture but the influence of these factors on other guilds were insignificant. The relationship between individual species and the above parameters were not analyzed due to the low number of observations. However this study shows that the dung age and moisture are not the only factors controlling the overall abundance and richness of dung beetles associated with elephant dung in the study region.

Four species are endemic to Western Ghats of which two (O. siva and Onthophagus madoqua) are specific to Nilgiri Hills and Onthophagus falsus is a new record from Western Ghats. The low abundance of Heliocopris in the elephant-rich study region compared to its high presence in other regions of Western Ghats (Joseph 1998) needs further study to reach conclusions. Heliocopris dominus, an exclusive feeder on elephant dung (Joseph 1998) is the ecological equivalent of Heliocopris dilloni that feeds on African elephant dung in east African savannahs (Halffter and Mathews 1966). Our enquiries with the resident tribal settlers and the resident forest officials confirmed that H. dominus is a common beetle straying into human dwellings attracted to light, in the wetter South Wayanad but rarely recorded from the study region in North Wayanad. This points towards the probability of regional variation in dung beetle distribution in Wayanad forests and comparative studies across different regions of the Western Ghats forests are necessary to interpret the variation in species composition of dung beetles associated with elephant dung.

The current study showed that the number of species and beetles associated with elephant dung pats is low in the region. The present study is an analysis of the dung pat beetle composition in deciduous forests during the post-rainy season for a limited time period of 21 days involving low sampling and covering a small geographical area. This low sampling effort might be a reason for the low observed species richness associated with elephant dung in this area. However, during this three-year field analysis of forest floor insect diversity of the region, bison dung pats attracted more dung beetles than elephant dung balls (unpublished personal observations). We speculate that this low presence of dung beetles in elephant pats is due to the probable preference of dung beetles (Holter *et al.* 2002) for the readily available more liquid dung pats of bison in the same region.

Differences in the diel activity pattern of beetle species in succession was not included in this study due to the practical difficulty in sampling during night, daybreak and twilight hours in the study region, which are infamous for frequent elephant-man confrontations. The moisture content of elephant dung depends on the diet of the elephant, the size of the dung heap, physical disturbance as well as the climate of the area (Anderson and Coe 1974). A complete round the year phenological survey considering the differences in diel activity of the beetles in relation to the shifting feeding pattern of elephants (Sukumar 2003) may provide more information on the community ecology of the dung beetles associated with the mega herbivores of this forest region.

## References

[i1536-2442-6-17-1-Anderson1] Anderson J. M. Coe M. J. 1974 Decomposition of elephant dung in an arid, tropical environment Oecologia 14 111 125 10.1007/BF00344902 28308101

[i1536-2442-6-17-1-Arrow1] Arrow G. J. 1931 The Fauna of British India including Ceylon and Burma, Coleoptera: Lamellicornia (Coprinae) i-xii 428 13 pls Taylor and Francis London

[i1536-2442-6-17-1-Balthasar1] Balthasar V. 1963a *Monographic der Scarabaeidae und Aphodiidae der Palaearktischen und Orientalischen Region (Coleoptera: Lamellicornia)*, 1 pp.391 PI. XXIV. Verlag der Tschechoslowakischen Akademie der Wissenschaften Prag

[i1536-2442-6-17-1-Balthasar2] Balthasar V. 1963b *Monographic der Scarabaeidae und Aphodiidae der Palaearktischen und Orientalischen Region (Coleoptera: Lamellicornia)*, 2 627 pp., PI. XVI. Verlag der Tschechoslowakischen Akademie der Wissenschaften Prag

[i1536-2442-6-17-1-Biswas1] Biswas S. Chatterjee S. K. 1986 Scarabaeidae (Coleoptera) of Silent Valley, Kerala, India, with description of three new species. Silent Valley special issue Records of the Zoological Survey of India 82 1–4 79 96

[i1536-2442-6-17-1-Biswas2] Biswas S. Chatterjee S. K. 1991 Fauna of Orissa. State Fauna Series 1 Zoological Survey of India 3 243 262

[i1536-2442-6-17-1-Biswas3] Biswas S. Mulay S. V. 2001 Fauna of Nilgiri Biosphere Reserve. Fauna of Conservation Area Series II Zoological Survey of India 129 142

[i1536-2442-6-17-1-Bray1] Bray J. R. Curtis C. T. 1957 An ordination of the upland forest communities of Southern Wisconsin Ecological Monograph 27 325 349

[i1536-2442-6-17-1-Brose1] Brose U. Martinez N. D. 2004 Estimating the species richness with variable mobility Oikos 105 292 300

[i1536-2442-6-17-1-Cambefort1] Cambefort Y. 1991 Dung beetles in tropical savannas In Hanski I, Cambefort Y, editors. *Dung beetle ecology* 156 178 Princeton University Press Princeton, New Jersey

[i1536-2442-6-17-1-Cambefort2] Cambefort Y. Hanski I. 1991 Dung beetle population biology In Hanski I, Cambefort Y, editors. *Dung beetle ecology* 37 50 Princeton University Press Princeton, New Jersey

[i1536-2442-6-17-1-Cambefort3] Cambefort Y. Walter P. 1991 Dung beetles in Tropical Forests in Africa In Hanski I, Cambefort Y, editors. *Dung beetle ecology* 198 210 Princeton University Press Princeton, New Jersey

[i1536-2442-6-17-1-Chatterjee1] Chatterjee S. K. Biswas S. 1995 Fauna of West Bengal. State Fauna Series 3 Zoological Survey of India 6 A 363 447

[i1536-2442-6-17-1-Chatterjee2] Chatterjee S. K. Biswas S. 2000 Fauna of Tripura State. Fauna Series 7 Zoological Survey of India 3 87 98

[i1536-2442-6-17-1-Chazdon1] Chazdon R. L. Colwell R. K. Denslow J. S. Guariguata M. R. 1998 Statistical methods for estimating species richness of woody regeneration in primary and secondary rain forests of northeastern Costa Rica In Dallmeier F, Comiskey JA, editors. *Forest biodiversity research, monitoring and modeling: conceptual background and old world case studies* 285 309 Parthenon Publishing Paris

[i1536-2442-6-17-1-Clarke1] Clarke K. R. Warwick R. M. 2001 *Change in marine communities: an approach to statistical analysis and interpretation* 2 PRIMER-E Plymouth

[i1536-2442-6-17-1-Colwell1] Colwell R. K. 2000 *EstimateS: statistical estimation of species richness and shared species from samples*. Version 6 User’s Guide and application available at: http://viceroy.eeb.uconn.edu/estimates

[i1536-2442-6-17-1-Colwell2] Colwell R. K. Coddington J. A. 1994 Estimating terrestrial biodiversity through extrapolation Philanthropical Transactions of Royal Society of London (Series B) 345 101 118 10.1098/rstb.1994.0091 7972351

[i1536-2442-6-17-1-Doube1] Doube B. M. 1991 Dung beetles of South Africa In Hanski I, Cambefort Y, editors. *Dung beetle ecology* 133 155 Princeton University Press Princeton, New Jersey

[i1536-2442-6-17-1-Feer1] Feer F. Pincebourde S. 2005 Diel flight activity and ecological segregation with in an assemblage of tropical forest dung and carrion beetles Journal of Tropical Ecology 21 21 30

[i1536-2442-6-17-1-Fisher1] Fisher R. A. Corbet A. S. Williams C. B. 1943 The relation between the number of species and the number of individuals in a random sample of an animal population Journal of Animal Ecology 12 42 58

[i1536-2442-6-17-1-Kerala1] *Forests and Wildlife Department working plan* 2001 North Wayanad Division, Forests and Wildlife Department Government of Kerala, India

[i1536-2442-6-17-1-Gause1] Gause G. F. 1934 The *struggle for existence* Baltimore 10.1126/science.79.2036.16-a 17821472

[i1536-2442-6-17-1-Gujarati1] Gujarati D. N. 2003 *Basic econometrics* Mc Graw- Hill, Inc

[i1536-2442-6-17-1-Halffter1] Halffter G. Mathews E. G. 1966 The natural history of dung beetles of the sub family Scarabaeinae (Coleoptera, Scarabaeidae) Folia Entomologica Mexicana 12–14 1 132

[i1536-2442-6-17-1-Hanski1] Hanski I. 1980 Patterns of beetle succession in droppings Annales Zoologici Fennici 17 17 25

[i1536-2442-6-17-1-Hanski2] Hanski I. 1991a The dung insect community In Hanski I, Cambefort Y, editors. *Dung beetle ecology* 5 21 Princeton University Press Princeton, New Jersey

[i1536-2442-6-17-1-Hanski3] Hanski I. 1991b Epilogue In Hanski I, Cambefort Y, editors. *Dung beetle ecology* 366 371 Princeton University Press Princeton, New Jersey

[i1536-2442-6-17-1-Hanski4] Hanski I. Cambefort Y. 1991 Competition in dung beetles In Hanski I, Cambefort Y, editors. *Dung beetle ecology* 305 329 Princeton University Press Princeton, New Jersey

[i1536-2442-6-17-1-Hanski5] Hanski I. Krikken J. 1991 Dung beetles in the Tropical forests of South-East Asia In Hanski I, Cambefort Y, editors. *Dung beetle ecology* 156 178 Princeton University Press Princeton, New Jersey

[i1536-2442-6-17-1-Heinrich1] Heinrich B. Bartholomew G. A. 1979 Roles of endothermy and size in inter- and intraspecific competition for elephant dung in an African dung beetle, Scarabaeus laevistriatus Physiological Zoology 52 484 96

[i1536-2442-6-17-1-Holter1] Holter P. 1982 Resource utilization and local coexistance in a guild of Scarabaeid dung beetles (Aphodius spp.) Oikos 9 213 227

[i1536-2442-6-17-1-Holter2] Holter P. Scholtz C. H. Wardhaugh K. G. 2002 Dung feeding in adult Scarabaeines (tunnellers and dwellers): even large dung beetles eat small particles Ecological Entomology 27 169 176

[i1536-2442-6-17-1-Joseph1] Joseph K. J. 1998 Biology and breeding behaviour of the elephant dung beetle, Heliocopris dominus Bates (Coprinae: Scarabaeidae) Entomon 23 325 329

[i1536-2442-6-17-1-Joy1] Joy M. S. 1991 *Keralathile Vanyajeevisankethangal* (Wild Life Reserves in Kerala) 194 State Institute of Languages Kerala

[i1536-2442-6-17-1-Kenderick1] Kenderick K. 1989 Sri Lanka In Campbell DG, Hammond HD, editors. *Floristic inventory of tropical countries.* The New York Botanical Garden New York

[i1536-2442-6-17-1-Hammond1] Hammond HD, editors. *Floristic inventory of tropical countries.* The New York Botanical Garden New York

[i1536-2442-6-17-1-Lobo1] Lobo J. M. Martin Piera F. Veiga C. M. 1988 Las trampas pitfall con cebo, sus posibilidades en el estudio de Scarabaeoidea (Col.).I. Caracteristicas determinantes de su capacidad de captura Revue D Ecologie Et De Biologie Du Sol 25 77 100

[i1536-2442-6-17-1-Magurran1] Magurran A. E. 2003 Measuring biological diversity Blackwell Publishing London

[i1536-2442-6-17-1-Mishra1] Mishra C. Johnsingh A. J. T. 1998 Population and conservation status of the Nilgiri tahr Hemitragus hylocrius in Anamalai Hills, South India Biological Conservation 86 199 206

[i1536-2442-6-17-1-Moore1] Moore I. 1954 An efficient method of collecting dung beetles Pan-Pacific Entomologist 30 208

[i1536-2442-6-17-1-Myers1] Myers N. 1988 Threatened biotas: 'hotspots' in tropical forestry The Environmentalist 8 1 20 10.1007/BF02240252 12322582

[i1536-2442-6-17-1-Nair1] Nair S. C. 1991 *The Southern Western Ghats-a biodiversity conservation plan.* Indian National Trust for Art and Cultural Heritage New Delhi

[i1536-2442-6-17-1-Oppenheiner1] Oppenheiner J. R. 1977 Ecology of dung beetles (Scarabaeidae: Coprinae) in two villages of West Bengal Records of the Zoological Survey of India 72 389 398

[i1536-2442-6-17-1-Pascal1] Pascal J. P. 1988 *Wet Evergreen Forests of the Western Ghats of India: Ecology, Structure, Florisitic Composition and Succession* French Institute Pondicherry

[i1536-2442-6-17-1-Peck1] Peck S. B. Howden H. F. 1984 Response of a dung beetle guild to different sizes of a dung bait in a Panamanian rainforest Biotropica 16 235 238

[i1536-2442-6-17-1-Rodgers1] Rodgers W. A. Panwar H. S. 1988 *Planning a wildlife protected areas network in India. Dept. of Environment, Forests, and Wildlife/Wildlife Institute of India report* 1 2 Wildlife Institute of India

[i1536-2442-6-17-1-Shorrocks1] Shorrocks B. 1990 Coexistence in a patchy environment In Shorrocks B, Swingland IR, editors. *Living in a patchy environment* 91 106 Oxford University Press

[i1536-2442-6-17-1-Simpson1] Simpson E. H. 1949 Measurement of diversity Nature 163 688

[i1536-2442-6-17-1-Smith1] Smith B. Wilson J. B. 1996 A consumer’s guide to evenness indices Oikos 76 70 82

[i1536-2442-6-17-1-Sowig1] Sowig P. Wassmer T. 1994 Resource partitioning in coprophagous beetles from sheep dung: phenology and microhabitat preferences Zool.Jb.Syst 121 171 192

[i1536-2442-6-17-1-Sukumar1] Sukumar R. 1989 *The Asian Elephant: Ecology and Management. Cambridge Studies in Applied Ecology and Resource Management* Cambridge University Press New York

[i1536-2442-6-17-1-Sukumar2] Sukumar R. 2003 *The living elephants* Oxford University Press

[i1536-2442-6-17-1-Toti1] Toti D. S. Coyle F. A. Miller J. A. 2000 A structured inventory of Appalachian grass bald and Heath Bald spider assemblages and a test of species richness estimator performance The Journal of Arachnology 28 329 345

[i1536-2442-6-17-1-Veiga1] Veiga C. M. Lobo J. M. Martin Piera F. 1989 Las trampas pitfall con cebo, sus posibilidades en el estudio de las comunidades de Scarabaeoidea (Col.). II. Analisis de efectividad Revue d'Ecologie et de Biologie du Sol 26 91 109

[i1536-2442-6-17-1-WCMC1] WCMC (World Conservation Monitoring Centre). *Global Biodiversity* 1992 Chapman and Hall London

[i1536-2442-6-17-1-WWF1] WWF Website 2001 *Wild world*, WWF full report, South Western Ghats montane rain forests (IMO151), Terrestrial Ecoregions web site: http://www.worldwildlife.org

[i1536-2442-6-17-1-Zar1] Zar J. H. 2003 *Biostatistical Analysis* Pearson Education (Singapore) Pte.Ltd., Indian branch Delhi, India

